# Nitrogen narcosis induced by repetitive hyperbaric nitrogen oxygen mixture exposure impairs long-term cognitive function in newborn mice

**DOI:** 10.1371/journal.pone.0196611

**Published:** 2018-04-26

**Authors:** Bin Peng, Shun-Hua Peng, Run-Ming Qu, Li-Hua Xu, Zheng-Lin Jiang

**Affiliations:** Department of Neurophysiology and Neuropharmacology, Institute of Nautical Medicine and Co-innovation Center of Neuroregeneration, Nantong University, Nantong, Jiangsu, China; Southeast University Zhongda Hospital, CHINA

## Abstract

Human beings are exposed to compressed air or a nitrogen-oxygen mixture, they will produce signs and symptoms of nitrogen narcosis such as amnesia or even loss of memory, which may be disappeared once back to the normobaric environment. This study was designed to investigate the effect of nitrogen narcosis induced by repetitive hyperbaric nitrogen-oxygen mixture exposure on long-term cognitive function in newborn mice and the underlying mechanisms. The electroencephalogram frequency was decreased while the amplitude was increased in a pressure-dependent manner during 0.6, 1.2, 1.8 MPa (million pascal) nitrogen-oxygen mixture exposures in adult mice. Nitrogen narcosis in postnatal days 7–9 mice but not in adult mice induced by repetitive hyperbaric exposure prolonged the latency to find the platform and decreased the number of platform-site crossovers during Morris water maze tests, and reduced the time in the center during the open field tests. An increase in the expression of cleaved caspase-3 in the hippocampus and cortex were observed immediately on the first day after hyperbaric exposure, and this lasted for seven days. Additionally, nitrogen narcosis induced loss of the dendritic spines but not of the neurons, which may mainly account for the cognitive dysfunction. Nitrogen narcosis induced long-term cognitive and emotional dysfunction in the postnatal mice but not in the adult mice, which may result from neuronal apoptosis and especially reduction of dendritic spines of neurons.

## Introduction

When human beings are exposed to compressed air or a nitrogen-oxygen mixture, they will produce nitrogen narcosis at a total pressure of three to four absolute atmospheres (ATA), exhibiting spatial and temporal disorientation, euphoria, hallucinations, disruptions in motor and locomotor coordination, and even loss of consciousness, which mainly depends on the partial pressure of nitrogen [1]. Hyperbaric nitrogen is considered to bind to specific hydrophobic sites or pockets within the membranous ion channels at the synapses (e.g., N-methyl-D-aspartate (NMDA) and gamma-aminobutyric acid (GABA) receptor channels), causing disruptions of inhibitory and excitatory synaptic function [2]. Studies in rats have shown that hyperbaric nitrogen induced changes in several extracellular striatal neurotransmitter levels, such as decreases in glutamate and dopamine release, disruptions of the regulation of the nigrostriatal pathway and desensitization of GABA receptors [3]. Additionally, the mechanisms of repetitive nitrogen exposures were different from the first exposure, which indicated that different effects on the brain induced by single or repetitive exposure may exist [4]. Previous studies have demonstrated that nitrogen narcosis causes decrements in memory performance through an impairment of input into long-term memory or of self-guided search and importantly a disruption of the encoding of information [5, 6]. However, the precise mechanism of memory impairment remains unclear.

Numerous studies have raised the possibility of an association between general anesthesia exposure and cognitive deficits (i.e., a brief or repetitive exposure to clinically relevant doses of commonly used anesthetics, including those that NMDA receptors inhibitors may cause an increase in anxiety-like behavior and a decrease in learning and memory function particularly in young and aged individuals) [7, 8]. The cognitive outcome depends on dose, exposure length and importantly, the developmental stage when general anesthesia is administered [9]. In the critical periods of development, experience-driven electrical activity patterns exert a crucial influence on neuronal network development, synaptic formation and synaptic contacts between cells. Thus, pharmacological interference such as general anesthesia can easily exert lasting effects on neuronal structure and function [9, 10]. Increasing experimental evidence shows that even short-term exposure to general anesthesia can induce long-term effects on neuronal networks [11].

Since similar symptoms and possibly the same targets are shared in nitrogen narcosis and in general anesthetics, the aim of this study was to investigate whether nitrogen narcosis will impair cognitive performance and emotion especially for those in developmental stages as general anesthetics do in humans and animals [9], and if so, what are the underlying mechanisms?

## Materials and methods

### Animals

A total of sixty-six adult male C57BL/6j mice (6–9 weeks of age) and ninety-two postnatal 7- days mice were provided by the Experimental Animal Center of Nantong University (Institutional license: SYXK(SU)-2012-0030). Totally sixteen adult mice randomly dividing into four groups (0 MPa, 0.6 MPa, 1.2 MPa, 1.8 MPa) were used to record the EEG changes, then the remaining fifty adult ones were randomly divided into five groups, which were control group, a single day or repeatedly for five, ten, or fifteen days. A total of ninety-two postnatal mice were randomly divided into two groups, which were 0 MPa and 1.8 MPa. All procedures involving animals were carried out in accordance with the Guidelines for the Care and Use of Laboratory Animals prepared by the Institutional Animal Care and Use Committee (IACUC) of Nantong University (NTU), Nantong, Jiangsu Province, China. All experimental protocols were reviewed and approved by the IACUC of NTU, approval number was 20140901–001. All surgery was performed under isoflurane anesthesia, and all efforts were made to minimize suffering. Mice were housed in a common cage and maintained on a regular day (06:00–18:00) /night (12 hours) cycle with free access to food and water. The temperature was maintained at 22 ± 1°C. Changes in food and water intake, fecal character, hair color, moving state, and body weight were monitored daily and no abnormalities were found.

### Hyperbaric exposure

Mice were exposed to hyperbaric mixed gas or normobaric air (Nantong Tianyuan Gas Co., Jiangsu, China) in a 100-liter chamber (Wuhu Diving Equipment Factory, Anhui, China) and the mice were placed in a cage and were free to move. The chamber pressure was increased at a rate of 100 kPa/s up to 200 kPa (additional pressure) using compressed air and then to 0.6, 1.2 and 1.8 MPa with pure nitrogen (Nantong Tianyuan Gas Co., Ltd., Jiangsu, China) at the same rate and was maintained at 1.8 MPa for three hours. The concentrations of oxygen and carbon dioxide were monitored in real time by SDA oxygen and carbon dioxide monitors (ANALOX, North Yorkshire, England), respectively. The partial pressure of oxygen was maintained manually at a constant value not exceeding 0.42 ATA during hyperbaric exposure while exhaled carbon dioxide was absorbed using soda lime. About a total of six hours was spent for decompression to the surface. Control animals were placed in the chamber ventilated with air and without compression to avoid stress involvement in the hyperbaric exposure-induced changes we investigated.

### Recording of electroencephalogram (EEG)

In brief, a total of sixteen adult mice were anesthetized with isoflurane, and then two bipolar stainless steel electrodes were planted into the forehead (2 mm after bregma, 2 mm lateral from the midline, and 1 mm deep under the skull) and the ground electrode was attached to prefrontal skin as described by JP, Wisor [12]. The surgery was performed on a heating pad. When the animals revived after surgery, each mouse was housed alone with free access to food and water. One week after the operation when the electrodes were completely fixed, EEG was monitored with an EEG recorder (RM6240BD, Chengdu Instruments Factory, Chengdu, China) while each mouse was caged and placed in the hyperbaric chamber. EEG was used to estimate the nitrogen narcosis, and both the frequency and amplitude of EEG were analyzed.

### Morris water maze test

The Morris water maze test was performed to evaluate the learning and memory function as MT Williams et al. described previously [13]. In brief, the learning trials were conducted over five days, with four trials per day. The latency to find the platform during the basic acquisition training was recorded and analyzed using AnyMaze software (Stoeling Co., Chicago, Illinois, USA). Spatial memory was assessed while the platform was removed as the number of platform-site crossovers in the target quadrant.

### Elevated O-maze test

The elevated O-maze and elevated plus-maze are two popular tests to assess the animal anxiety level. In the present study, we used the elevated O-maze to evaluate the changes in the anxiety levels of adult mice after ten-day repetitive exposures. Different with plus-maze, the elevated O-maze lacks a center square but consists of an annular runway (width 5.5 cm, outer diameter 46 cm, 50 cm above ground level) and two open and two closed segments. The closed segments are enclosed by walls extending 20 cm above the surface of the maze while there are no walls in the open segments. This device was placed in an isolated room away from any extraneous interference including noise, scent, movement, and etc. The animals were placed in one of the protected sectors and observed for 8 min while the duration and frequency of entries into the open or closed segments were recorded by a camera using AnyMaze software (Stoeling Co., Chicago, Illinois, USA) [14].

### Open field test

The open field test was also used to assess the anxiety level of mice subjected to hyperbaric exposure during its development stage after completion of the Morris water maze test [15]. It consists of a square arena (50×50 cm, 50 cm height), with a white floor divided into two square regions, i.e., central zone and peripheral zone. The test was initiated by placing a mouse in the corner of the arena and letting it move freely for 30 min. Mouse activity was continuously videotaped by a video camera placed over the device. The arena was carefully cleaned with alcohol and rinsed with water after every test. Finally, the total time spent in the center during the total exploration time was recorded by a camera using AnyMaze software (Stoeling Co., Chicago, Illinois, USA).

### Western blotting analysis

Since the hippocampus and cortex are the important regions of learning and memory function, these two areas were quickly harvested from mice killed by decapitation under isoflurane anesthesia immediately on the1^st^ day (P10), 3^rd^ day (P12) and 7^th^ day (P16) after hyperbaric exposure, homogenized in Radio Immunoprecipitation Assay Lysis Buffer (Beyotime, Nantong, Jiangsu, China), centrifuged at 14,000 g at 4°C for 30 minutes, the supernatant was then collected, and the total protein concentration was determined using a BCA Protein Assay Kit (Thermo Scientific, Rockford, Alabama, USA). Protein samples mixed with loading buffer were electrophoresed using 10% sodium lauryl sulfate-polyacrylamide gel electrophoresis and electrically transferred onto a polyvinylidene fluoride membrane. The membrane was incubated at 4°C overnight in tris buffered saline containing 5% defatted milk and detected with the following primary antibodies: rabbit anti-cleaved caspase-3 polyclonal antibody (1:1000; #9662; CST Co., Boston, Massachusetts, USA), rabbit anti-Bax (1:1000; #14796; CST Co., Boston, Massachusetts, USA), rabbit anti-Bcl-2 (1:1000; #2876; CST Co., Boston, Massachusetts, USA), mouse anti-β-actin (1:8000; #A5316, Sigma-Aldrich Co., St. Louis, Missouri, USA). After several washes in Tris-buffered saline, secondary IRDye 800 CW goat anti-mouse or rabbit (1:10000, Li-COR, Lincoln, Nebraska, USA) was incubated for two hours at room temperature, and the immunoreactivities were captured using a fluorescence scanner (Odyssey Lix, Li-COR, Lincoln, Nebraska, USA). Semi-quantitative evaluation of protein levels was performed by densitometric scanning using Image-pro Plus 5.1 software (Media Cybernetics, Bethesda, Maryland, USA) and the data are presented as percentages relative to the control samples assumed to be 100%.

### Immunohistochemistry

Mice were anesthetized with isoflurane and then perfused with 50 mL of normal saline through the left ventricle followed by a 50 mL 4% paraformaldehyde solution after decompression. The brain tissue was removed quickly and then post-fixed for 48 hours in 4% paraformaldehyde solution. The brains were serially cut into 25 μm-thick coronal sections from the same region, and then diaminobenzidine (DAB) staining was performed. In brief, sections were incubated at 4°C overnight with rabbit anti-cleaved caspase-3 polyclonal antibody (1:1000; #9662; CST Co., Boston, USA), followed by incubation with a secondary antibody (Goat anti-Rabbit, 1:200, F9887, Sigma-Aldrich Co., St. Louis, Missouri, USA) for one hour; the sections were then incubated with DAB (D5637, Sigma-Aldrich Co., St. Louis, Missouri, USA) solution until brown products appeared under the microscope. Finally, the sections were examined under a microscope (DM 4000B, Leica, Wetzlar, Hesse-Darmstadt, Germany), and then quantitated as the percentage (caspase-3 positive cells / Nissl positive cells) with Image-pro Plus 5.1 software (Media Cybernetics, Bethesda, Maryland, USA).

### Nissl staining

Nissl staining was used to observe the number and morphology of neurons. Brain tissue was prepared as above and coronal sections were cut with a thickness of 5 μm on a paraffin microtome (RM2245, Leica, Bensheim, Hesse, Germany) after paraffin embedding. Brain sections were then deparaffinized, rehydrated, stained with cresyl violet, mounted with neutral balata, and covered with coverslips. Finally, the sections were examined under a microscope (DM 4000B, Leica, Wetzlar, Hesse-Darmstadt, Germany).

### Golgi staining

Golgi staining was used to observe the density of neuronal dendritic spines. Briefly, brain tissue was quickly removed under isoflurane anesthesia and placed in Golgi impregnation buffer for two weeks according to the protocol described in the FD rapid Golgi Stain kit (PK 401/401A, Version 2014–02, FD Neuro Technologies Inc., Columbus, Ohio, USA). Golgi-Cox-stained brains were then cut into cross-sections of 100 μm thickness with a Vibratome (VT1000S, Leica, Bensheim, Hesse, Germany), and pictures were captured using a microscope (SP8, Leica, Wetzlar, Hesse, Germany) and analyzed using Image-Pro Plus software (Media Cybernetics, Bethesda, Maryland, USA).

### Statistical analysis

All the data are presented as the mean ± S.E.M. Student’s *t*-test was used for independent samples, and one-way analysis of variance (ANOVA) or two-way ANOVA with hyperbaric exposure and time (repeated measure) as factors were used for multiple comparisons (LSD) while the variables were normally distributed after testing with Shapiro-Wilk Test Kolmogorov-Smirnov (*P* > 0.05) using SPSS 17.0 software. The significance level was established at *P* < 0.05.

## Results

### Effects of hyperbaric nitrogen-oxygen mixtures exposure on EEG in adult mice

**Fig 1:**
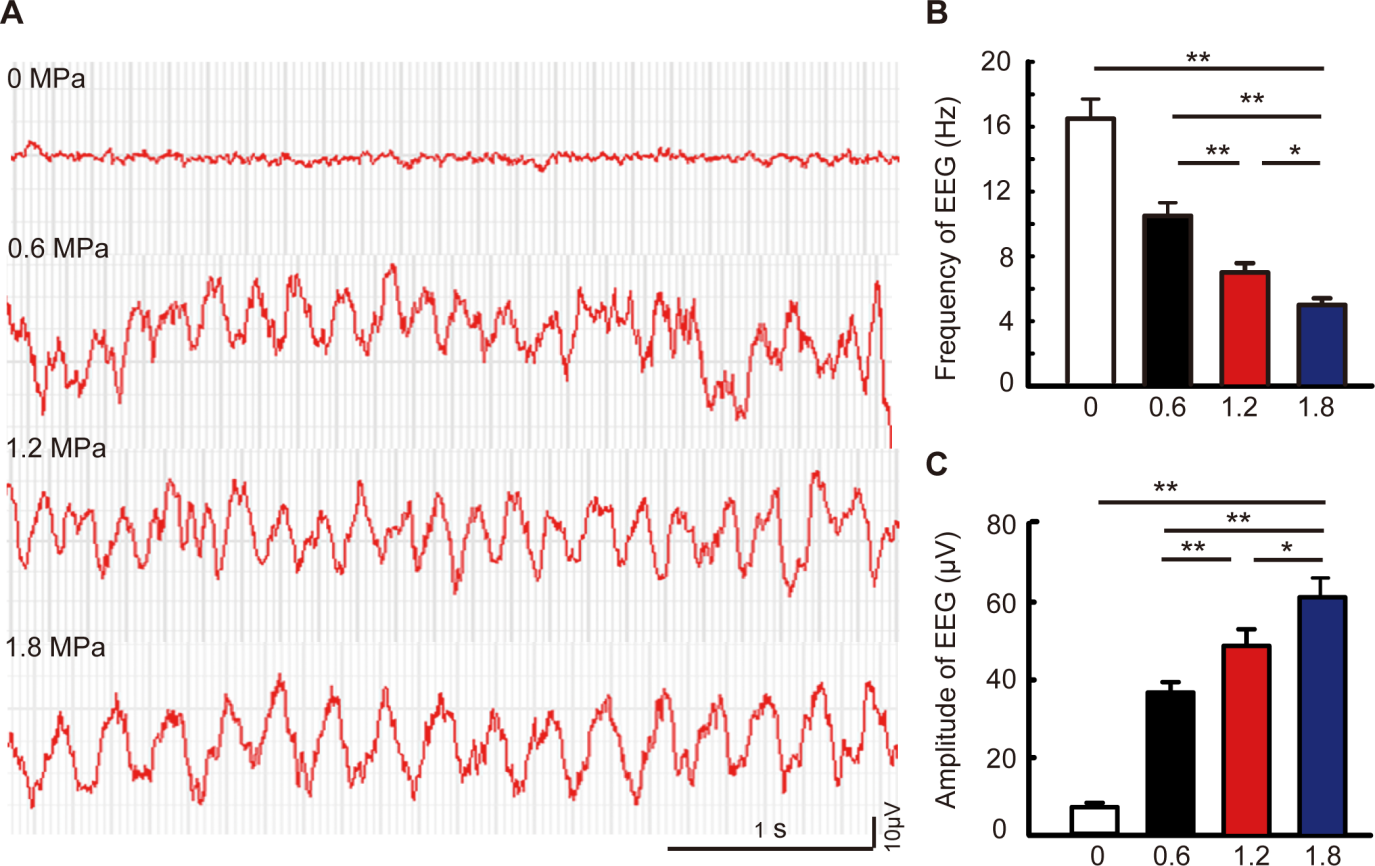
EEG changes of adult mice exposed to the increased pressure of nitrogen-oxygen mixtures. **(A)** Representative dynamic EEG changes at 0 MPa, 0.6 MPa, 1.2 MPa, and 1.8 MPa groups during hyperbaric exposure. **(B and C)** The frequency and the amplitude of EEG in each group, respectively, n = 4/group, *P < 0.05, **P < 0.01.

To detect the narcotic potency of nitrogen in mice, we monitored the dynamic changes of EEG during 0.6, 1.2, and 1.8 MPa hyperbaric nitrogen-oxygen mixture exposure. We found that there were mainly fast waves (alpha 8–12 Hz and beta 13–30 Hz) under normal pressure (Fig 1A). However, a slow-wave rhythm occurred (delta 1–3 Hz and theta 4–7 Hz) during hyperbaric nitrogen exposure (Fig 1A), and the frequency was significantly decreased (Fig 1B, one-way ANOVA, LSD post-hoc test, *F*_3, 15_ = 55.455, *P* < 0.01, n = 4/group) while the amplitude was remarkably increased in a pressure-dependent manner (Fig 1C, one-way ANOVA, LSD post-hoc test, *F*_3, 15_ = 47.562, *P < 0*.*01*, n = 4/group). Since changes in the EEG frequency and amplitude during 1.8 MPa hyperbaric nitrogen-oxygen mixture exposure were most prominent, our subsequent experiments focused on 1.8 MPa hyperbaric exposure.

### Repetitive hyperbaric nitrogen exposures in adult mice exerted no significant influence on cognitive function and anxiety behavior

**Fig 2:**
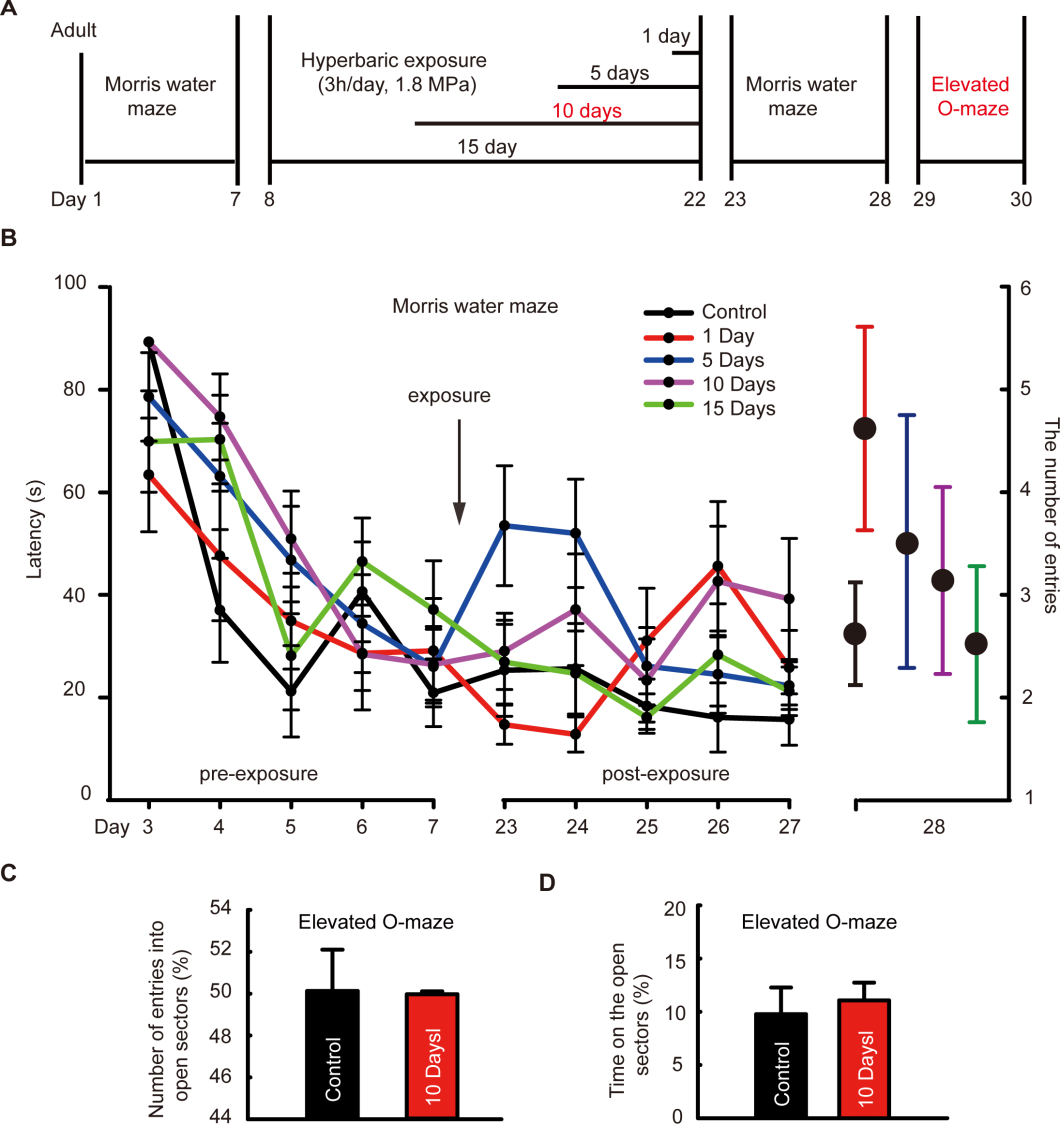
Effects of single or repetitive 1.8 MPa nitrogen-oxygen mixture exposure on learning and memory function and anxiety behavior in adult mice. **(A)** Concise experiment protocol of adult mice. **(B)** The latency and the number of platform-site crossovers (the number of entries) in the Morris water maze, n = 10/group. **(C and D)** The number of entries into open sectors/the total entries and the time spent in the open sectors /total time spent in elevated O-maze tests, n = 10/ group.

To investigate the after-effect of nitrogen narcosis on learning and memory function and anxiety behavior, adult mice were exposed to hyperbaric nitrogen-oxygen mixtures for a single day or repeatedly for five, ten, or fifteen days (Fig 2A). In the Morris water maze tests, the latency of mice to find the platform was decreasing as time went on (Fig 2B, influence of time, two-way ANOVA with repeated measurements, *F*_9, 17_ = 28.824, *P* < 0.01, n = 10/group), but non-significant influence of time × hyperbaric exposure interaction was found among groups (Fig 2B, two-way ANOVA with repeated measurements, *F*_36, 160_ = 3.354, *P* = 0.106, n = 10/group); At Day 28, there was no significant difference in the number of platform-site crossovers among groups in the Morris water maze (Fig 2B, one-way ANOVA, *F*_4, 79_ = 1.36, *P* = 0.2629, n = 10). To further observe the effect of repetitive exposures on anxiety behavior, we detected the number and the time of entries into open sectors using elevated O-maze test in the ten-days repetitive exposure group, but no statistical significances were found while compared to control group (Fig 2C, *t*_18_ = 0.07, *P* = 0.945; Fig 2D, *t*_18_ = -0.478, *P* = 0.639, n = 10/group).

### Repetitive hyperbaric nitrogen exposures in postnatal days 7–9 mice impaired learning and memory function and induced an increase in anxiety-like behavior at adulthood

**Fig 3:**
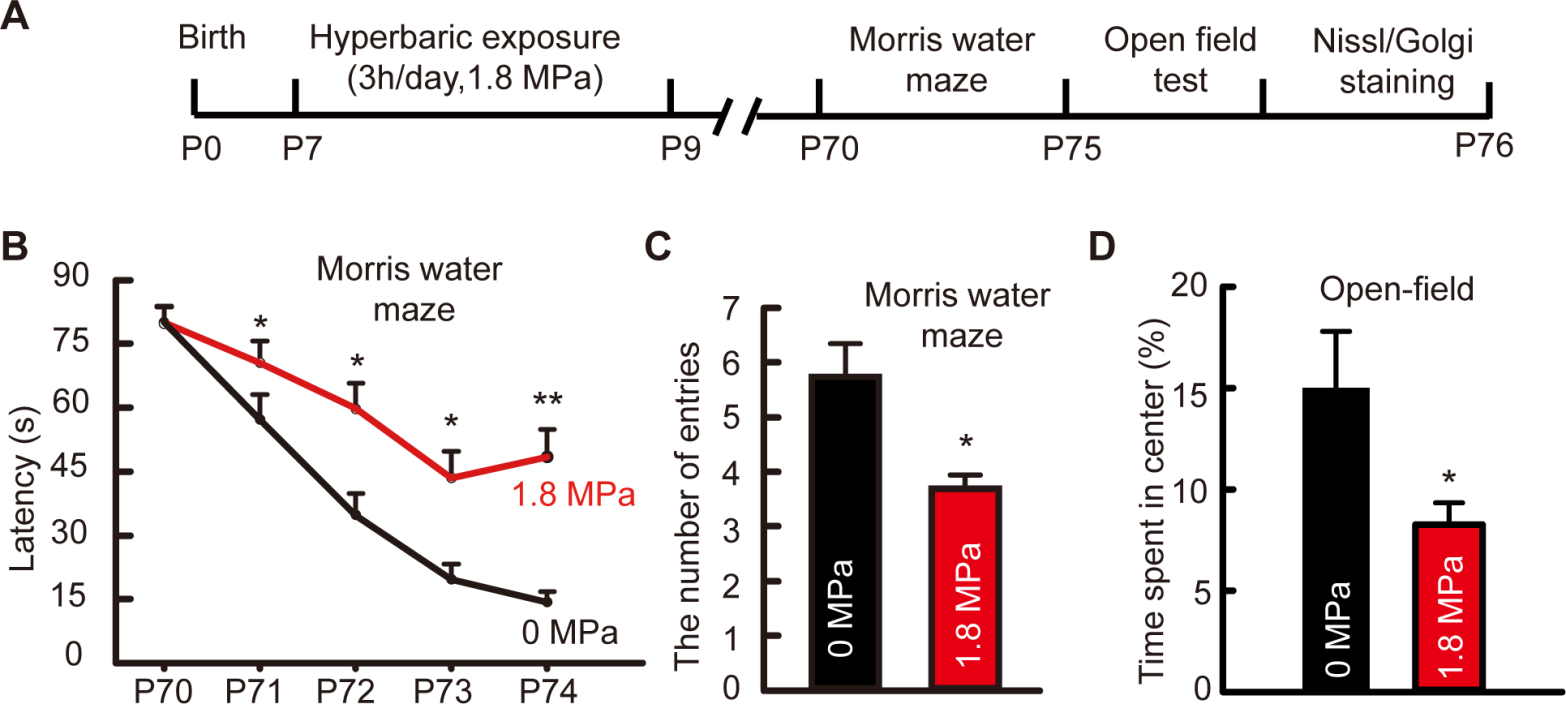
Effects of repetitive exposure in postnatal mice on long-term learning and memory function and anxiety behavior. **(A)** Experiment protocol of postnatal mice in Fig 3 and Fig 6. **(B and C)** The latency and the number of platform-site crossovers (the number of entries) in the Morris water maze, *P < 0.05, **P < 0.01 *vs*. 0 MPa group, n = 8/group. **(D)** The anxiety behavior indicated by the time spent in the center during the open-field test. *P < 0.05 *vs*. 0 MPa group, n = 8/group.

In the Morris water maze, less time were spent to find the platform as the time went on (Fig 3B, influence of time, two-way ANOVA with repeated measurements, *F*_4, 56_ = 38.343, *P < 0*.*01*, n = 8/group), and significant influence of time × hyperbaric exposure interaction was found (Fig 3B, two-way ANOVA with repeated measurements, LSD post-hoc test, *F*_4, 56_ = 2.572, *P < 0*.*05*, n = 8/group), while the number of platform-site crossovers was decreased after repetitive hyperbaric exposures (Fig 3C, *t*_14_ = 2.9012, *P* < 0.05, n = 8/group). Mice receiving repetitive 1.8 MPa hyperbaric exposures early at postnatal days 7–9 spent less time in the center compared to that in control group during open field test (Fig 3D, *t*_14_ = 2.266, *P* < 0.05, n = 8/group).

### Repetitive hyperbaric nitrogen exposures induced neural cell apoptosis in the hippocampus and cortex of postnatal days 10–16 mice

**Fig 4:**
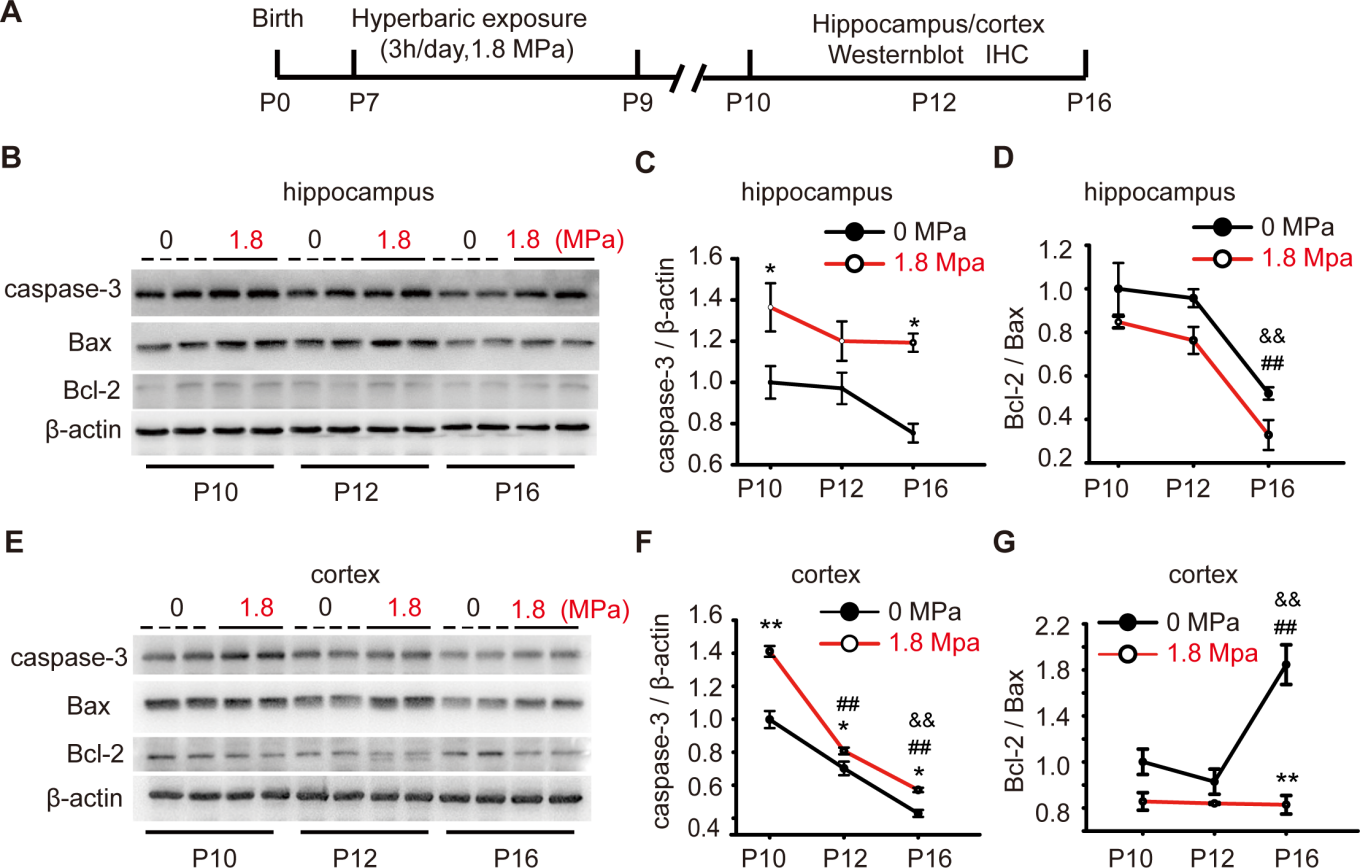
Hyperbaric exposure induced neural cell apoptosis in the brain of postnatal mice. **(A)** Experiment protocol of postnatal mice in Figs 4 and 5. **(B-G)** Representative immunoblotting pictures and quantitative analysis for cleaved caspase-3, Bax and Bcl-2 on the 1^st^ day (P10), 3^rd^ day (P12) and 7^th^ day (P16) after hyperbaric exposure, n = 6/group, *P < 0.05, **P < 0.01 *vs*. 0 MPa group, ^##^*P* < 0.01 *vs*. 1^st^ day (P10), ^&&^*P* < 0.01 *vs*. 3^rd^ day (P12).

**Fig 5:**
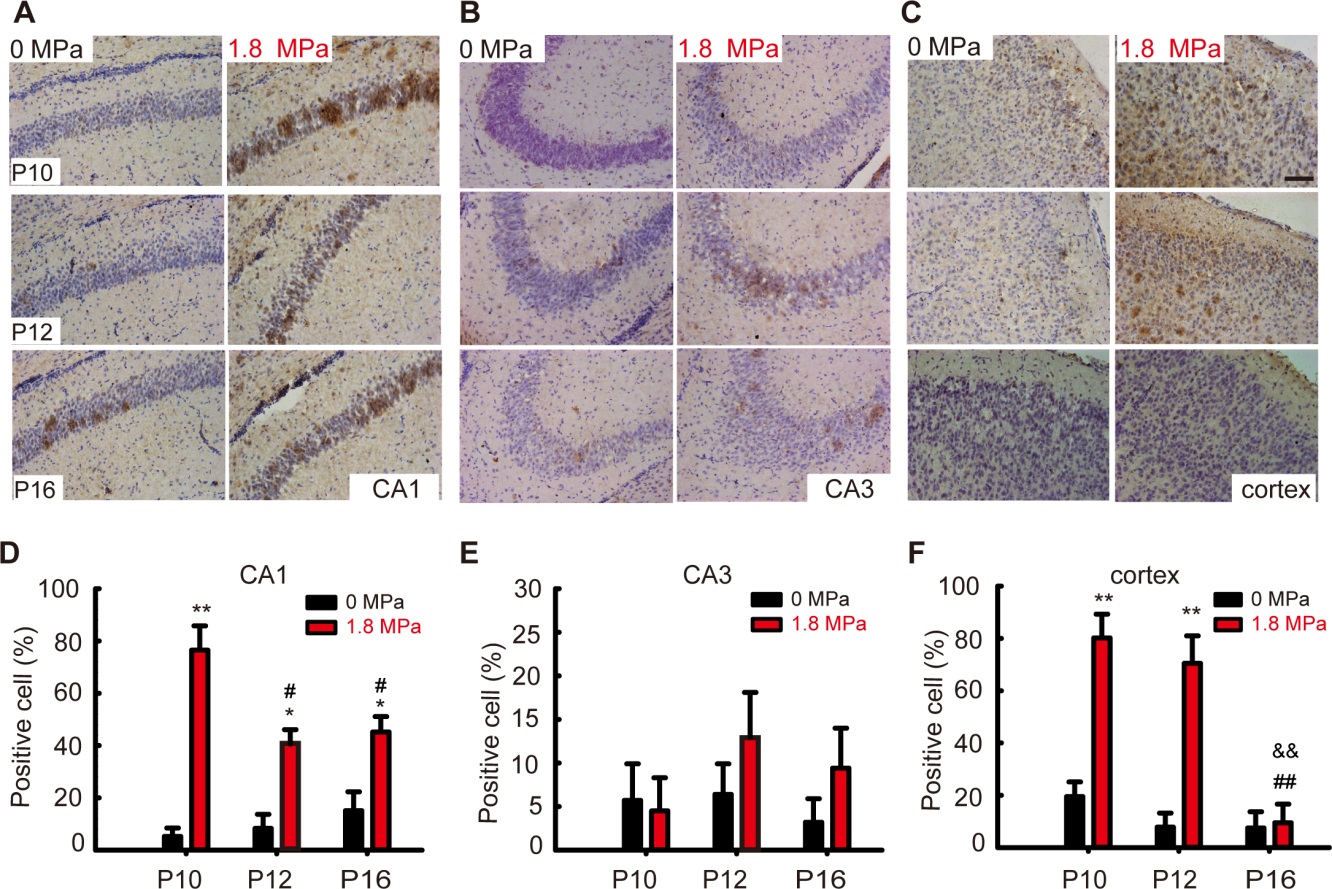
The expression and distribution of caspase-3 positive cell in the brain of postnatal mice after repetitive hyperbaric exposures. **(A-C)** Representative immunohistochemistry pictures of cleaved caspase-3 in the hippocampal CA1 and CA3 areas and in the cortex, scale bar = 100 μM. **(D-F)** The counting of caspase-3 positive cell, *P < 0.05, **P < 0.01 *vs*. 0 MPa group, ^#^*P* < 0.05 *vs*. 1^st^ day (P10), ^&&^*P* < 0.01 *vs*. 3^rd^ day (P12), n = 6/group.

To examine the potential mechanism related to the behavioral abnormalities caused by repetitive hyperbaric nitrogen exposures, we determined the levels of apoptosis-related proteins on the 1^st^ day (P10), 3^rd^ day (P12) and 7^th^ day (P16) after hyperbaric exposure (Fig 4A). The cleaved caspase-3 level was increased both in the hippocampus (*P* < 0.05, Fig 4B and 4C, n = 6/group) and cortex (*P* < 0.05 or 0.01, Fig 4E and 4F, n = 6/group) immediately on the 1st day after hyperbaric exposure, lasting until the 7th day we observed, except at P12 in the hippocampus (*P* > 0.05, Fig 4C). In addition, the ratio of Bcl-2 and Bax expression (Bcl-2/Bax) was decreased at P12 in the cortex (*P* < 0.01, Fig 4E and 4G, n = 6/group) after hyperbaric exposure. Using immunohistochemistry, we found that the percentage of caspase-3 positive cells in CA1 areas after hyperbaric exposure was increased when compared to that in the control group (Fig 5A and 5D, one-way ANOVA, LSD post-hoc test, *F*_5, 35_ = 18.66, *P* < 0.01, n = 6/group). No significant differences were observed in CA3 areas among groups (Fig 5B and 5E, one-way ANOVA, *F*_5, 35_ = 0.96, *P* = 0.585, n = 6/group). In the cortex, a number of positive cells were seen at P10 and P12 in exposure groups (Fig 5C and 5F, one-way ANOVA, LSD post-hoc test, *F*_5, 35_ = 19.93, *P < 0*.*01*, n = 6). All these results suggested that apoptosis induced by hyperbaric exposure mainly occurred in the CA1 areas of the hippocampus and in the cortex.

### Repetitive hyperbaric nitrogen exposures early in postnatal days 7–9 mice did not induce long-term neuronal loss but reduced dendritic spine densities in the hippocampus and cortex at adulthood

**Fig 6:**
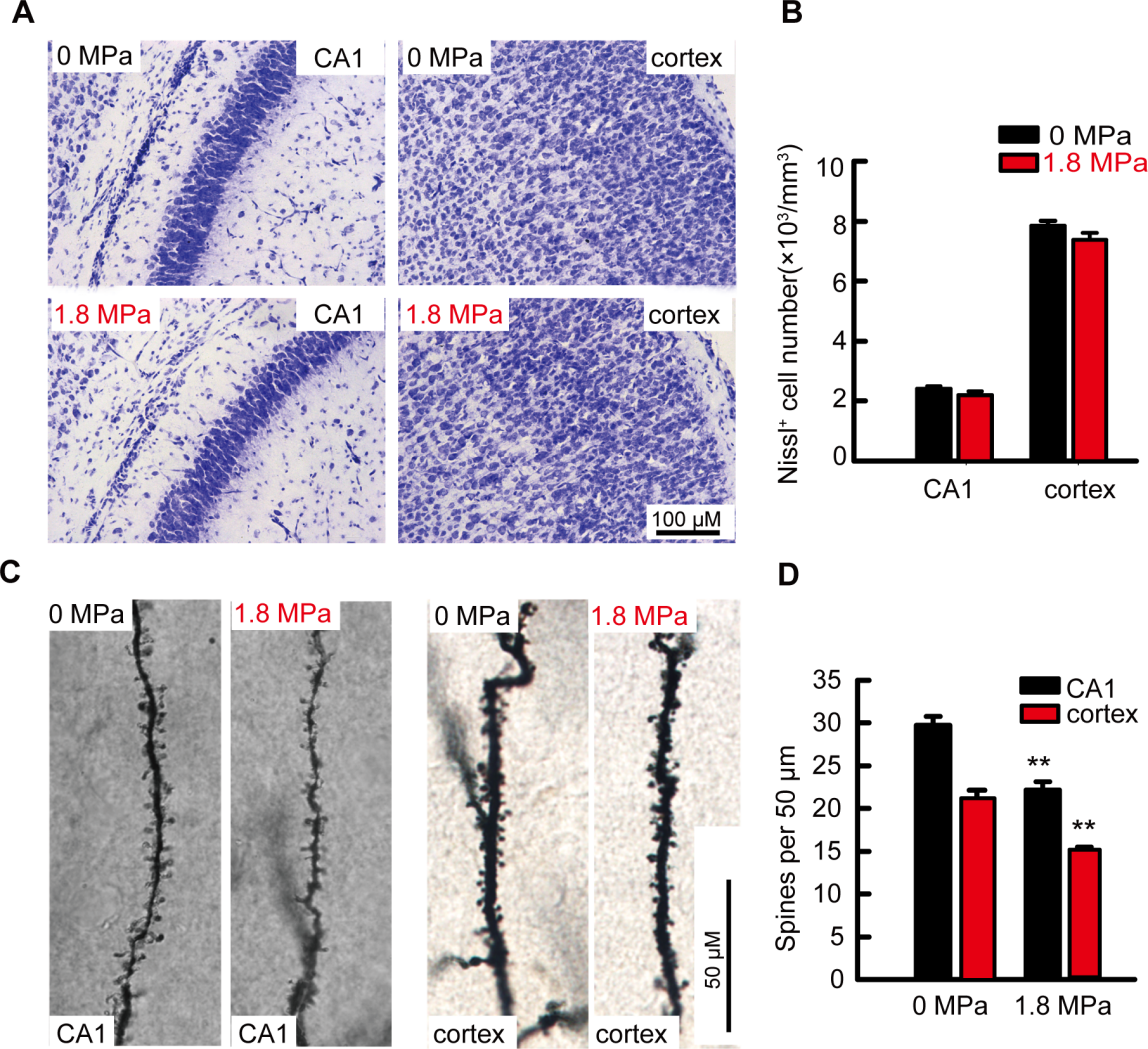
Repetitive exposure of hyperbaric nitrogen-oxygen mixtures in postnatal mice induced long-term loss of dendritic spines but did not affect the number of neurons. **(A)** Representative Nissl staining of CA1 and cortex at the adult stage, scale bar = 100 μM. **(B)** Counting of positive cells, n = 6/group. **(C)** Representative Golgi staining pictures in CA1 and cortex, scale bar = 50 μM. **(D)** Counting of dendritic spines, n = 4/group, **P < 0.01 *vs*. 0 MPa group.

Since neural cell apoptosis occurred immediately after hyperbaric nitrogen exposure, we further observed the long-term effect of hyperbaric exposure at the early postnatal stage on the number of neurons and the density of dendritic spines in the brain at the adult stage. Interestingly, we did not find significant neuronal loss in the CA1 (*t* = 1.216, *P* = 0.291) areas of the hippocampus and in the cortex (*t* = 1.729, *P* = 0.159) (Fig 6A and 6B, n = 6/group); however, the density of dendritic spines of neurons both in the hippocampal CA1 areas (*t* = 5.385, *P* < 0.01, n = 4/group) and in the cortex (*t* = 8.333, *P* < 0.01, n = 4/group) were decreased after repetitive hyperbaric nitrogen exposure (Fig 6C and 6D).

## Discussion

Nitrogen narcosis is a reversible change in consciousness, neuromuscular function, and behavior brought on by breathing compressed air or nitrogen-oxygen mixtures [16]. The symptoms begin first with impairment of the higher function including judgment, reasoning, short-term memory, and concentration. Some divers may also experience a euphoric or stimulating feeling initially which is similar to mild alcohol intoxication. Further increase in the nitrogen pressure while descending deeper leads to impairments in motor coordination and even in sensory and conscious functions such as manual dexterity and further mental decline, which are similar to those from general anesthetic [1]. Neurophysiological changes include slowing of rhythms in the EEG (more theta and delta activity) and differences in cortical evoked potentials [16, 17]. Similar to nitrogen, many other inert gases such as neon, argon, krypton, and xenon can also cause an anesthetic effect. Up till now, the exact mechanism of inert gas narcosis has not been fully elucidated, more experiments support the protein theory which involves neurotransmission, herein NMDA or GAGA receptors are the main targets [16, 18], both of which are implicated in general anesthesia especially induced by gaseous anesthetics [18]. There is still debate whether repetitive hyperbaric nitrogen exposures will induce long-term impairments on the central nervous system (CNS) [19–21]. It is generally acknowledged that inert gas narcosis completely resolves upon ascent. It poses no problem in the long term and does not lead to chronic issues or predispose to increased or decreased susceptibility to recurrent exposures [16]. However, we do not know whether inert gas narcosis will induce long-term problem on the CNS in children especially in critical periods of development as the general anesthetics do [9–11].

Although nitrogen narcosis is reported for all mammals, the thresholds in men and rodents differ greatly (e.g., the signs and symptoms appear in men at 0.3 MPa and at 1 MPa in rats) [22]. In the present study, when mice were exposed to hyperbaric nitrogen-oxygen mixtures, the frequency on EEG was decreased while the amplitude was increased in a pressure-dependent manner, indicating a nitrogen narcosis in mice at this condition. This result is in line with previous studies that reported an increase in slow waves on EEG (delta and theta) during hyperbaric nitrogen exposure [17, 23].

Experimental evidence has demonstrated that exposure to general anesthetics will induce long-term morphological and functional changes in the CNS that in turn may impair cognitive performance, especially in young children and aged people [7, 9]. However, no impairment of memory was found in adult mice after single or repetitive exposure in the present study. How does it act in young bodies? We further measured the behavioral performance in young mice that received hyperbaric nitrogen exposure early at critical stages of development. In this study, when postnatal mice were consecutively exposed to hyperbaric nitrogen from P7-P9, anxiety behavior was increased and learning and memory functions were impaired, which were in accordance with findings from general anesthesia [24–27], suggesting a long-term behavioral and cognitive dysfunction after repeated hyperbaric nitrogen exposures. In the developing brain, neurogenesis in the hippocampal dentate gyrus represents an actively ongoing form of developmental morpho-functional plasticity that persists into adulthood, which makes it easy to be interfered by nitrogen narcosis [10]. Thus, in the present study, long-term impairments of the cognitive and emotional functions were detected in the postnatal mice but not in the adults.

Some studies have revealed that exposure to general anesthetics can rapidly trigger cell apoptosis and death in the CNS during brain region-specific developmental time windows in the developing brain [8, 28]. The peak of apoptotic response was observed early at 3 h after anesthesia exposure, and the apoptosis degree depended on the dose and exposure length [29]. In this study, a wide range of cell apoptotic response was also found both in the hippocampus and cortex early at the first day and lasted at least for seven days. We observed after consecutive hyperbaric exposure what has occurred with general anesthesia. Both early-life short- and long-term anesthesia exposure induced apoptosis in neurons, but only the long-term exposure was associated with lasting cognitive deficits [30]. The evidence proving the causal link between early-life general anesthesia and lasting cognitive dysfunction is not sufficient, but many experimental works support this possibility [9]. Thus, the cell apoptosis we observed immediately after hyperbaric exposure at an early age provides a potential cause underlying the long-term learning and memory dysfunction, as well as the abnormal anxiety behavior in mice [26, 31, 32]. In our study, however, there were no significant changes in the number of neurons in hippocampus and cortex, which may result from relatively low degrees of cell apoptosis as occurs following isoflurane inhalation [33]. Nevertheless, we found a decrease in the number of dendritic spines in the hippocampal CA1 area and cortex at adulthood after 3 times of hyperbaric nitrogen exposure at postnatal mice (P7-P9) in this study. In line with our observations, even short-term general anesthetic exposure could induce a marked loss in the number of dendritic spines and synapses as early as a couple of hours and importantly persist into adulthood [34]. Furthermore, in the developing brain, many researchers consider that it is the long-term changes in synaptic connectivity rather than apoptosis that are responsible for cognitive dysfunction [9]. The present data imply that nitrogen narcosis may impair cognitive function during developmental stages through targeting synaptic formation and loss. However, several fundamental mechanisms including neurotrophin signaling, mitochondrial function and tau phosphorylation regarding the long-term effects of nitrogen narcosis on the CNS remain unresolved [9]. It is apparently necessary to design further studies to understand the mechanisms underlying the long-term effects of nitrogen narcosis on the CNS.

In conclusion, the present study found that hyperbaric nitrogen exposure in postnatal mice exerted long-term impairment in cognitive and emotional functions, but these effects were not in the adult mice, which may result from neuronal apoptosis and especially reduction of dendritic spines of neurons.

## Supporting information

S1 File The tab of animal experimental ethical inspection. (PDF)
