# Supplementary material for: Nitrogen narcosis induced by repetitive hyperbaric nitrogen oxygen mixture exposure impairs long-term cognitive function in newborn mice
Source: PLoS One. 2018 Apr 26;13(4):e0196611. doi: 10.1371/journal.pone.0196611 (PMC5919656; doi:10.1371/journal.pone.0196611)
Supplement: S1 File — (PDF) [file pone.0196611.s001.pdf]

动物实验伦理审查表

The Tab of Animal Experimental Ethical Inspection

编号(No): 20140901-001

申请人填写的相关信息 (Concerned information wrote by applicant)

申请单位 (Name of organization)：南通大学航海医学研究所 (Institute of Nautical Medicine, Nantong University)

申请人姓名 (Name of applicant)	学历 (Education)	技术职称(Professional title)	岗位证书编号 (Certificate No.)
彭彬 (Peng Bin)	硕士 (Master)	助理研究员 (Research assistant)	2100266

实验名称(Study title)：重复高压氮氧混合气暴露诱导的氮麻醉对新生小鼠远期认知功能的影响 (Nitrogen Narcosis Induced by Repetitive Hyperbaric Nitrogen Oxygen Mixture Exposure Impairs Long-term Cognitive Function in Newborn Mice)

实验目的(Aim of experiment)：探讨重复的高压氮氧混合气暴露诱导的氮麻醉对新生小鼠远期认知功能的影响及机制(Investigate the effect of nitrogen narcosis induced by repetitive hyperbaric nitrogen-oxygen mixture exposure on long-term cognitive function in newborn mice and the underlying mechanisms)

拟进动物情况

动物来源(Source of animal)：南通大学实验动物中心(Laboratory Animal Center of NTU)

品种品系(Species or strain)： C57BL/6j 等级(Grade)： SPF

规格(Specifications)： 7 days and 6-9 weeks of age

数量(Number)： 190	申请日期(Application date)：2014/09/01<br>2014 年 9 月 1 日
进驻日期(Entering date)：2015/09/15<br>2014 年 9 月 15 日	结束日期(Ending date)：2015/04/05<br>2015 年 4 月 5 日

实验要点,包括实验方法、观测指标、实验结束后处死动物的方法等(Outline of experiments, experimental methods, observational index, executing animal method et. al)：

实验方法(experimental methods)

高压氮氧混合气暴露 （Hyperbaric Nitrogen-Oxygen Mixture Exposure）

观测指标(observational index)

脑电图（EEG），莫里斯水迷宫（Morris Water Maze），旷场试验（Open-Field），高架O迷宫（Elevated O-Maze）

处死动物(executing animal method)

异氟烷麻醉 （under isoflurane anesthesia）

南通大学实验动物中心标准操作规程

The Standard Operating Procedures for Laboratory Animal Center of NTU

<table>
<tr><td></td><td colspan="5">申请人签名 (Signature of applicant): [signature] 联系电话 (Telephone): +86-0513-55003331</td></tr>
<tr><td>审查依据
(Inspection contents)</td><td colspan="5">1. 该项目是否必须用实验动物进行实验，即能否用计算机模拟、细胞培养等非生命方法替代动物或用低等动物替代高等动物进行实验(Does laboratory animal must be used in the project? Could other methods such as computer simulation, cell cultivation or using the low-grade animal instead of the high-grade animal)？

2. 表中所填申请人资格和所用动物的品种品系、质量等级、规格是否合适，能否通过改良设计方案或用高质量的动物来减少所用动物的数量(Are the qualification of applicant，species or strain, grade and specifications of animals suitable? Could the quantity of animals be reduced by improving the study design or using high quality animals)？

3. 能否通过改进实验方法、调整实验观测指标、改良处死动物的方法，来优化实验方案、善待动物(Could the study design and animal treatment be refined by ameliorating experimental method, adjusting observational index, executing animal method)？</td></tr>
<tr><td rowspan="3">审查结果
(是否同意申请人的实验方案)
(Results of inspection)</td><td>课题负责人意见
(Study director):</td><td>同意
(Agree) ☑</td><td>不同意
(Disagree) ☐</td><td>签名
(Signature)</td><td>[signature]</td></tr>
<tr><td>实验动物中心主任意见
(Director of animal experimental center):</td><td>同意
(Agree) ☐</td><td>不同意
(Disagree) ☐</td><td>签名
(Signature)</td><td>[signature]</td></tr>
<tr><td>实验动物伦理委员会意见
(The Lab Animal Ethical Committee):</td><td>同意
(Agree) ☑</td><td>不同意
(Disagree) ☐</td><td>签章(Stamp)</td><td>南通大学 实验动物中心 [stamp]</td></tr>
<tr><td colspan="6">备注(Supplement):</td></tr>
</table>
